# ‘Language is the source of misunderstandings’–impact of terminology on public perceptions of health promotion messages

**DOI:** 10.1186/s12889-015-1884-1

**Published:** 2015-06-23

**Authors:** Christina H. Buckton, Michael E. J. Lean, Emilie Combet

**Affiliations:** Human Nutrition, School of Medicine, College of Medical Veterinary and Life Sciences, University of Glasgow, Glasgow Royal Infirmary, Alexandra Parade, Glasgow, G31 2ER UK

**Keywords:** Health promotion, Public health, Policy, Diet, Food choice, Nutrition

## Abstract

**Background:**

The high level of premature death due to non-communicable diseases has been associated with unhealthful lifestyles, including poor diet. The effectiveness of public health strategies designed to promote health via messages focusing on food and diets depends largely on the perception of the messages by the public. The aim of this study was to explore public perceptions of language commonly used to communicate concepts linking health, food and the diet.

**Methods:**

This study is a qualitative and semi-quantitative cross-sectional survey exploring public perceptions of terms used to improve eating habits within public health strategies. We recruited adults with no background in nutrition or health-care, from May to July 2013, from urban areas of varying deprivation (*n* = 12) in Glasgow and Edinburgh, UK. Four key prompt-terms used to convey the idea of improving health through diet were selected for testing: *Healthy Eating*, *Eating for Health*, *Balanced Diet* and *Nutritional Balance*. Consumer understanding of these terms was explored using mixed-methods, including qualitative focus groups (*n* = 17) and an interviewer-led word-association exercise (*n* = 270).

**Results:**

The word-association exercise produced 1,386 individual responses from the four prompt-terms, with 130 unique responses associated with a single term. Cluster analysis revealed 16 key themes, with responses affected by prompt-term used, age, gender and socio-economic status. *Healthy Eating* was associated with foods considered ‘healthy’ (*p* <0.05); *Eating for Health* and *Balanced Diet* with negative connotations of foods to avoid (both *p* <0.001) and *Nutritional Balance* with the benefits of eating healthily (*p* <0.01). Focus groups revealed clear differences in perceptions: *Eating for Health* = positive action one takes to manage existing medical conditions, *Healthy Eating* = passive aspirational term associated with weight management, *Balanced Diet* = old fashioned, also dieting for weight loss, *Nutritional Balance* = maximising physical performance. Food suppliers use *Healthy Eating* terminology to promote weight management products. Focus group participants welcomed product reformulation to enhance food health properties as a strategy to overcome desensitisation to health-messages.

**Conclusions:**

Public perceptions of messages communicating concepts linking health, food and the diet are influenced by terminology, resulting in confusion. To increase individual commitment to change eating habits in the long term, public health campaigns need strengthening, potentially by investing in tailored approaches to meet the needs of defined groups of consumers.

**Electronic supplementary material:**

The online version of this article (doi:10.1186/s12889-015-1884-1) contains supplementary material, which is available to authorized users.

## Background

Since the early 1980s, governments worldwide have made substantial efforts to address the contribution of poor diets to high levels of disability and premature death from non-communicable diseases (NCDs) [1]. Scotland is a good example, with higher rates of NCDs than most other European Countries [2] and well-documented variations in social deprivation, diet-quality and health [3]. The Scottish Government has made considerable efforts to improve the quality of the diet, with the 1996 comprehensive national nutrition policy document “Eating for Health: A Diet Action Plan for Scotland” setting out a number of targets to be met by 2005. None of these targets were met, except for a reduction of total fat as a percentage of energy intake from around 40 % to 38 % (the target was 35 % or less) [4]. A study monitoring dietary habits in Scotland from 2001 to 2009 concluded that insufficient changes had taken place to have a significant impact on public health [5]. There are no examples of effective long-term national nutrition policies elsewhere [6].

A high-quality diet is based on a selection of nutrient-rich foods to match the reference intake of all nutrients (approximating the dietary needs for optimal health) without exceeding the reference energy intake (with further cultural and economic considerations, e.g. seasonality, level of processing, provenance). Policy measures designed to promote high-quality diets and dietary behaviours supportive of health fall into two main categories: information/educational measures designed to support informed consumer choice, and policy measures designed to change the market or physical environment. Information measures are easier to implement, but have a limited record of success, with raised awareness not necessarily translating into changed consumer behaviour [7]. Measures targeting the market or physical environment eg: fiscal measures, regulation (for example of school meals), physical access to food and reformulation, are more controversial for industry, politically and for freedom of choice [8] but may be more effective [9]. Several challenges emerge in relation to the implementation of food reformulation by industry: i) current reformulation strategies almost exclusively focus on nutrients that are in excess in the diet (i.e. energy, fat, salt and sugars), ii) the emphasis is therefore on weight control and limiting nutrients negatively associated with health, rather than the provision of a balance of nutrients positively associated with health and disease prevention, and iii) reformulated foods may or may not be acceptable to consumers [10].

Behaviour change has been well studied in relation to health [11–14], and requires individuals to have the capability (e.g. cooking skills, knowledge, income), the opportunity (e.g. physical access to healthy food, time) and the motivation (health status, interest in and understanding of consequences) to change. There is a complex interplay between these drivers of behaviour and the interventions designed to influence them [14]. Members of the public are exposed, daily, to a large volume of messages related to food and health from a range of sources with varying levels of reliability and consistency [15–17], leading to the desensitisation of consumers to messages linking food, eating and health [18, 19].

There is a need to define and understand public perceptions of these messages and the role and impact of the language and terminology used. Work in this area has focused on nutritional labelling, mostly linked to nutritional knowledge [20–22], and perception of nutrition and health claims [23]. Using construal theory, Ronteltap et al. highlighted that the concepts surrounding eating and health are not clear for consumers, and are not understood and interpreted identically by all [24]. The popular expression ‘healthy eating’ does not convey the notion of long-term influence on health and is often confused with, or used to refer to weight management, or dieting for weight loss [25, 26]. A further concern involves the perceived authenticity and credibility of ‘health messages’. Food product marketing has found value in using ‘healthy eating’ terminology and may, to some extent, dilute or confuse genuine health promotion [27, 28].

This study explored public perceptions around the terminology commonly used for health promotion in UK and other English language regions in relation to the concepts linking health, food and the diet, and examined demographic and social influences, making use of the wide variations found in Scottish urban settings. The specific research questions explored are:

1. What do common terms used for health promotion, focusing on diet and foods, mean to consumers?
2. Do these terms convey different meanings, and are there other terms that would communicate the concepts linking diet, food and health more clearly?
3. What are public perceptions of government policies designed to promote health via the diet and foods, including product reformulation?

## Methods

The study was conducted during the period May-July 2013 in accordance with the guidelines laid down in the Declaration of Helsinki. All volunteers provided implied informed consent to participate in the word association exercise, and written informed consent for participation in the focus groups. All procedures were approved by the University of Glasgow College of Medicine, Veterinary Medicine and Life Sciences (MVLS) Ethical Committee.

### Selection of key prompt-terms

Terms commonly used for health promotion focusing on diet and foods were identified by a search of the literature using databases PubMed and Web of Knowledge. Key search terms used were: (health* AND eat*) AND (perception* OR belief* OR attitude*). Additionally, ‘healthy’ food ranges carried by an indicative sample of 8 large supermarkets were reviewed, to determine the language used to market this concept (Table 1). Four terms commonly used for health promotion focusing on diet and foods were selected: ‘*healthy eating*’, ‘*eating for health’*, ‘*balanced diet*’ and ‘*nutritional balance*’. The four terms were piloted for recognition by a sample of the public attending the Glasgow Science Festival before inclusion as prompt-terms in this study.

**Table 1:** Language used by an indicative sample of large supermarkets to communicate the concept of ‘healthy options’ as opposed to ‘diet ranges’ (product ranges aimed at people seeking weight loss/management)

Supermarket or brand	Name of product range	Type of product
Sainsbury’s	Deliciously Balanced	Healthy option
	My Goodness	Diet range
Tesco	Light Choices	Diet range
	Healthy living	Healthy option
Waitrose	Love life – you count…	Diet range
	Perfectly balanced (now withdrawn)	Healthy option
Marks & Spencer	Delicious & Nutritious	Healthy option
	Fuller for Longer	Diet range
Eat Balanced	Nutritionally balanced	Healthy option
Safeway	Eating right	Diet range
Asda	Good for you	Diet range
Morrisons	NuMe	Diet range
Co-op	Healthier choice	Diet range

### Semi quantitative exploration of public reactions to key terms using word-association

Adults with no background in nutrition or healthcare, were recruited from 12 locations in Edinburgh and Glasgow. These areas were selected to represent a range of socio-economic areas as defined by the Scottish Index of Multiple Deprivation [29]. To eliminate researcher bias, a standard intercept survey technique was used to approach participants [30]. The researcher was stationed at a particular point at each recruitment location and approached every person passing a specific landmark. Eligible participants were shown one of the four prompt-terms, in strict rotation, using standard cards. They were then asked to say out loud the first six words that came to their mind, without any prompting from the researcher. These words were recorded on data capture forms along with information about the location, date and key demographic data: age, gender, and the first four digits of the participant’s home postcode.

### Focus groups and in-depth analysis of public perceptions

Participants (same criteria as above) were recruited from across Edinburgh and Glasgow using convenience and snowballing sampling, after advertising the study online [31]. Those taking part were eligible to enter a prize draw for vouchers as a reward. There was no pre-defined limit to the number of focus groups planned, with the intention of reaching theoretical saturation [32]. Focus groups were facilitated by the same researcher using a standard topic guide and sequence of questions to ensure consistency between groups [33] (Additional file 1). Full transcripts were manually coded by the researcher and organised into themes [34]. The topic guide structure was used as the first level with further sub-themes identified within each main theme.

### Data analysis and statistics

Analyses were conducted using IBM SPSS Statistics version 19.0.0 for Mac. Descriptive statistics included summaries of gender, age and deprivation level.

Response frequencies were analysed by gender, age, level of deprivation and prompt-term used. Age and level of residential deprivation determined through the DepCat Carstairs score based on the 2001 Scottish Census [35], were collapsed into two categories: younger (<45 years) and older (= >45 years) and low (DepCat scores 1–3) and high (DepCat scores 4–7).

Cluster analysis [36] was conducted on the list of responses generated by the word-association exercise to produce key themes, using a manual researcher-generated coding scheme. An initial set of themes was identified and an iterative process used to ensure all individual responses were categorised into the appropriate cluster. The Pearson *χ*^2^ test was used to test statistical significance in the patterns of these themes elicited by the prompt-term used, gender, age and deprivation level. Where the number of expected terms in a cell was below five, Fisher’s exact test was used.

## Results

### Participants

Two hundred seventy people participated in the word-association exercise across the 12 recruitment locations in Edinburgh (*n* = 63) and Glasgow (*n* = 207). This sample size affords a confidence interval (margin of error) of ±6 % for data reported, at the 95 % confidence level [37]. Males (*n* = 144, 53 %) and females (*n* = 126, 47 %) were equally represented, and 63 % were aged less than 45 (median age 36, range 69). Most (66 %) fell into the higher level of socioeconomic deprivation category (median deprivation category 4, range 6). Demographic characteristics were similar for the Edinburgh and Glasgow samples, with lower levels of deprivation for the Edinburgh sample (median 3, range 6 versus median 4, range 6, respectively) (Additional file 2).

Four in-depth focus groups were held, three in Glasgow and one in Edinburgh. Participants (*n* = 17) were mostly female (65 %), with a median age of 42 (range 44), with most (82 %) residing in areas of higher socioeconomic deprivation (median deprivation category 5, range 6). Participation in the focus groups was low, as the study required participants not sensitised to the subject matter; it was therefore challenging to generate high levels of interest. Demographics varied between the four focus groups for gender, age and level of socioeconomic deprivation, in order to attain the widest range of views possible. Efforts were made to ensure consistency within each focus group to ensure open and easy conversation.

### Research question 1: What do common terms used for health promotion, focusing on diet and foods, mean to consumers?

**Fig. 1:**
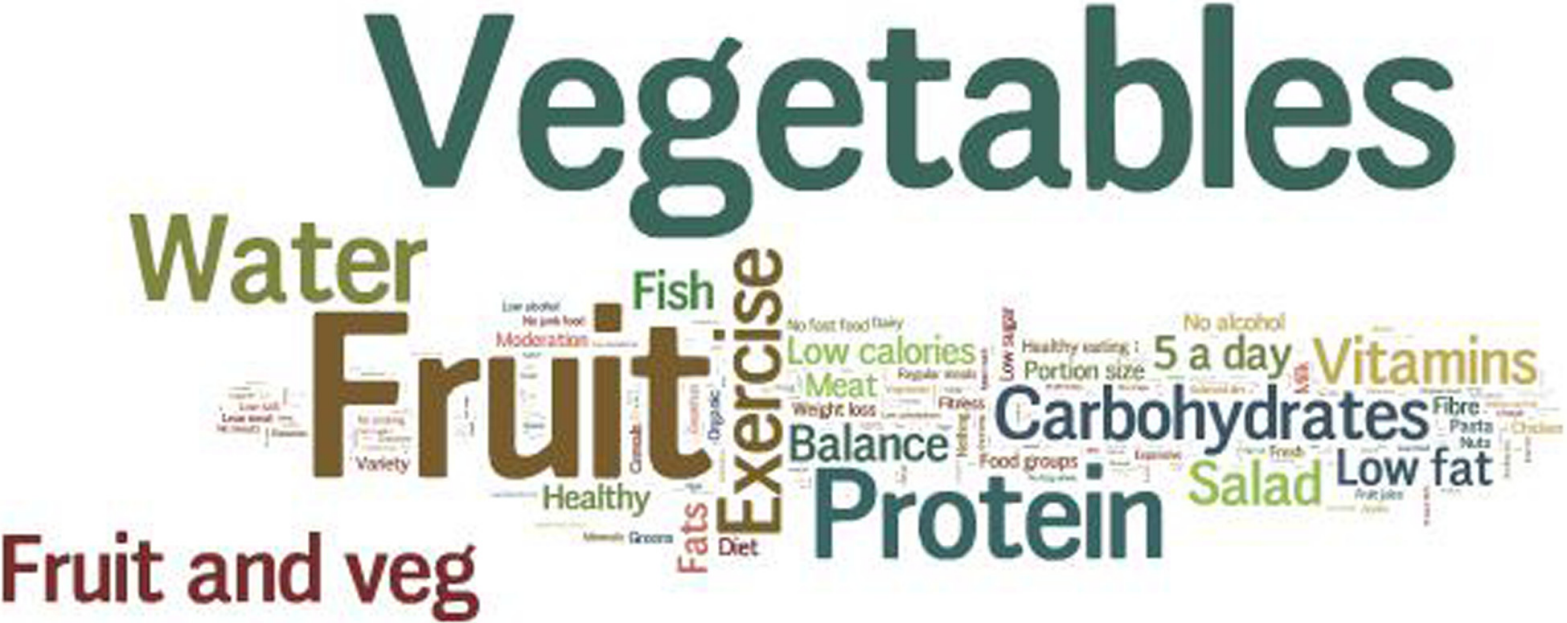
Word cloud illustrating the frequency of responses in the word association exercise. The more frequently a response was given, the larger its representation in the cloud

The word-association exercise produced 1,386 individual responses across the four prompt-terms, 260 of which were unique. The five most frequent responses for all four terms were vegetables, fruit, protein, water and ‘fruit & vegetables’ (Fig. 1) [38]. The twenty most frequent responses accounted for almost 55 % of all responses, while 118 were cited once only (8 % of all responses) (Table 2). The least commonly cited responses included mentioning specific micronutrients or food items and some personal views such as ‘*government bullshit*’ and ‘*I don’t eat for health*’.

**Table 2:** Top 20 most frequently given unique responses in word-association exercise

		Most frequent		
		%	n	Cum %
1	Vegetables	9	121	9
2	Fruit	8	114	17
3	Protein	5	62	22
4	Water	4	60	26
5	Fruit & vegetables	3	46	29
6	Exercise	3	44	32
7	Carbohydrates	3	36	35
8	Vitamins	2	32	37
9	Salad	2	30	39
10	Fish	2	26	41
11	Low fat	2	25	43
12	Balance	2	25	45
13	5 a day	2	23	47
14	Fats	1	20	48
15	Low calorie	1	19	49
16	Meat	1	18	50
17	Healthy	1	17	51
18	Portion size	1	14	52
19	Fibre	1	12	53
20	Food groups	1	12	54

Results are pooled for all four prompt-terms

Cum, Cumulative percentage

Cluster analysis of the 260 unique responses produced 16 key themes (Table 3). The largest cluster described foods perceived as ‘healthy’ and included 46 % of all responses. The second largest cluster described food-components to be avoided such as fat, sugar, and salt (12 %). Over 85 % of participants gave at least one response in the theme foods that are considered to be ‘healthy’ and 34 % responded with foods that should be avoided. The smaller clusters revealed some negative connotations with the concept of healthy eating such as ‘*Reasons to have to do it*’, ‘*nothing came to mind*’ and ‘*I don’t do it*’. Some examples of reasons given not to eat for health were due a lack of understanding of what it actually means and the perception that it is expensive, boring, and unsatisfying.

**Table 3:** Sixteen themes identified from cluster analysis of responses elicited during the word-association exercise with total responses for each theme (including duplicated responses), number of unique responses for each theme and proportion of the participants giving at least one response for each theme

		Total responses		Unique responses^a^		By participant^b^	
		%	n	%	n	%	n
1	Foods considered to be healthy	46	639	27	71	86	232
2	Foods that should be avoided	12	159	15	40	34	91
3	Descriptions of macronutrients	9	119	7	19	27	74
4	Characteristics of healthy foods	7	93	9	22	26	71
5	Balance and moderation	6	83	2	4	23	63
6	The importance of lifestyle	6	80	6	15	25	67
7	Benefits of doing it	3	46	7	19	12	31
8	Importance of micronutrients	3	44	3	7	14	38
9	Reasons not to do it	3	41	10	26	10	27
10	Diet and weight management	2	31	5	12	10	26
11	Food groups	1	18	1	3	6	16
12	Reasons to have to do it	1	14	4	11	4	12
13	Nothing came to mind…	0.5	7	0.8	2	3	7
14	I don’t do it	0.4	6	1	4	1	3
15	It’s just something I do	0.2	3	0.8	2	1	3
16	It’s very important	0.2	3	1	3	1	3
	Total		1,386		260		764

^a^ Unique responses eliminates duplicated responses

^b^ Indicates number of the 270 participants with at least one response in that theme

The focus groups highlighted the interpretation of the concept of *Healthy Eating* as meaning eating for weight loss or weight management. All four groups understood ‘healthy’ food choices to mean a consideration of the fat, calorie, sugar and salt content of food (Table 4). Half (53 %) of the participants directly stated that they do not consider health when making food choices, although they recognised that this depended on circumstances such as age (younger people were less interested in the associations between diet and long term health), financial situation (eating healthily was seen as expensive), culture (particularly family background) and the impact of an existing medical condition; those who knew people with conditions such as diabetes and high blood pressure said they paid more attention to their diet.

**Table 4:** Summary of main discussion areas arising from in-depth focus groups with illustrative examples of comments made during the discussions

Main themes	Sub-themes	Sample focus group quotes
1. Health as a consideration when making food choices	• Healthy means low calorie/fat	*A3: “I do so more when I’m on a diet, so I notice things like calories and fat.”*
	• Lack of interest	*D1: “I don’t care about my health, I’m young and want to try new things. I can worry about that in the future.”*
		*A3: “I want something that I really like, I don’t care even if the calories are there or if it’s balanced or not.”*
		*C5: “You see I never think about that, and I’ve got high blood pressure as well. I just shove salt on everything, I absolutely love salt.”*
	• Weight management	*B3: “…and the other reason is not to get fat. I don’t want to get fat, so I try to eat healthily for that reason.”*
	• Managing an existing condition	*A5: “I think it’s very hard for people to stick to a diet unless they have disease or some problem. Only those people can stick to a certain type of food or healthy food. For normal people I think it is very hard.”*
	• Depends on circumstances	*A4: “I don’t go out for meals very often but when I do, I’m just going to eat whatever I fancy from the menu…”*
		*D2: “Me, a lot, although I go through phases, like in the last few weeks I’ve not really done it”*
	• Financial considerations	*A4: “I’m trying to save my pennies so I wouldn’t chose carrots with the organic label.”*
	• Impact of culture	*B3: “When I was growing up we didn’t have much choice, we just had to eat whatever was put on the table.”*
		*C2: “The problem is we socialise so much now.”*
	• Control	*B3: “I don’t eat things like sweets or cakes, I don’t want to get used to them cause they’re tasty and they’re not healthy.”*
		*C2: “A lot of it’s to do with food, it just seems like every day is a fight.*
	• Use of supplements	*C1: “I don’t look at the vitamin or mineral content of food, but I would take a supplement, I just think it’s your insurance policy – you just take one and you know you’ve got it covered.”*
2. Influence of branding products as ‘healthy options’ on food choices	Confusion with diet products	*A1: “I think that sometimes food packages say low calories and people equate that with healthy eating.”*
		*C5: “So if I wanted to buy something like that, I would be thinking that was a kind of low fat option.”*
	• ‘Healthy’ options are tasteless	*A2: “No I don’t, I’ve tried low calorie, and low fat and I’ve just found them tasteless. I’d rather have a decent meal that fills me up.”*
	• ’Healthy’ options are no healthier	*C1: “The ‘good’ ones are no better than the other ones they sell. When they test them, they find they’re not all that.”*
		*C4: “Yes I would go for the healthier option, if it really was healthier.”*
Views on government policies designed to promote health via the diet and foods (information leaflets, dietary guidelines and reformulation)	• Awareness	*A2: “I’ve never seen it no. It's not something, even if I had noticed it, I might have glanced at it maybe, but I wouldn't pay any attention to it. It’s just not of interest.”*
		*D3: “Maybe I’m not as curious as I should be about finding out about what a balanced diet is.”*
	• Usefulness	*A3: “Even if you explained it to me I’d be like, oh that’s very nice, but I wouldn't do anything about it.”*
		*C2: “How would you work out a third, is it by weight?”*
		*D2: “I’m browsing through it and there’s too much text, I think it needs more graphics.”*
	• Nutritional labelling	*B4: “Nope, I don’t look at them.”*
		*C1: “Occasionally I will look, to make sure it’s not too fattening.”*
	• Reformulation	*D2: “I think free choice is a good argument but at the same time people aren’t actually that strong, or they want their small pleasures and don’t care what happens to them.”*
		*C2: “Then we’d eat two biscuits instead of one cause there’s less calories!”*
		*A1: “It would depend on price, if I walked into the supermarket and it was the most expensive thing I wouldn’t get it.”*
3. Views on the terminology used to communicate concepts linking health, food and the diet	• Perceived differences	*B4: “They all mean the same thing, if you’re having a balanced diet then you’re eating healthily and you’re eating for your health and it’s all nutrients isn't it?”*
		*C5: “All the same, it’s much of a muchness isn't it?”*
	• Eating for health	*A1: “No I actually think they are different. Eating for health would give you the idea that you had some kind of condition and you’d researched what you should eat for that condition.”*
		*D2: I don't know, that one sounds kind of weird to me.”*
		*D3: “… that one has a negative feel to me, it just feels like too much hard work.”*
	• Healthy eating	*A4: “I don’t know, healthy eating is more passive somehow.”*
		*D1: “Healthy eating is also like a general thing, it’s just about what you’re eating.”*
	• Balanced diet	*B1: “This one is good for me, if I want to lose weight.”*
		*A3: “Balanced diet means that I do eat nutritionally balanced food but the portions are smaller.”*
	• Nutritional balance	*C3: “For me that is more for like sport, who need nutritional balance to make sure they obtain optimum performance.”*
		*D2: “…you’d aim it at the market that’s interested is sport and going to the gym.”*
	• Suggestions for other terminology	No other suggestions made

### Research question 2: Do these terms convey different meanings, and are there other terms that would communicate the concepts linking diet, food and health more clearly?

**Fig. 2:**
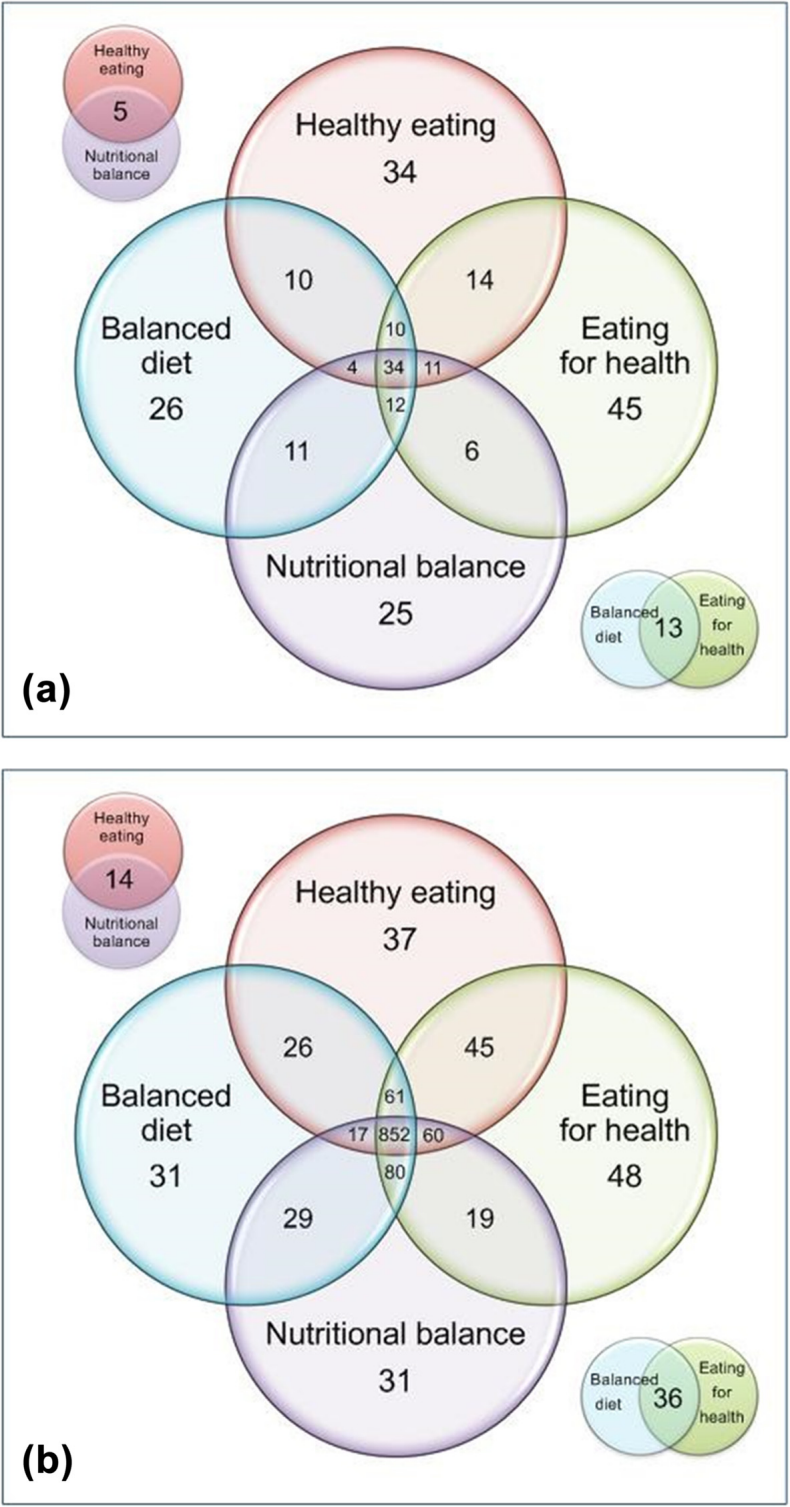
Venn diagrams illustrating overlap and differences in responses produced by the four terms tested (**a**) unique responses only (*n* = 260), (**b**) all responses (*n* = 1,386)

In the word-association exercise, use of the four different prompt-terms produced different patterns of response as well as the overlap between the terms. *Healthy Eating* produced *n* = 34 unique responses, *Eating for Health n = −*45, *Balanced Diet n =* 26 and *Nutritional Balance n =* 25 (Fig. 2).

Significantly different patterns of response were observed across the 16 key themes when analysed according to gender, age, deprivation category of participants and prompt-term, using Pearson’s *χ*^2^ test of association or Fisher’s exact test as appropriate (Table 5). Females were more likely to focus on foods perceived to be ‘healthy’ (*p* <0.05). Only men stated that they did not *Eat for Health* (*p* <0.05). Younger people were less likely to talk in terms of foods perceived to be’ healthy’ (*p* <0.05) or foods to avoid (*p* <0.001) and more likely to consider food groups (*p* <0.05) than older people. Older people were more likely to say that they do not eat for health (*p* <0.05). Finally those living in areas of greater socioeconomic deprivation were more likely to talk about foods that should be avoided (*p* <0.01), and the reasons not to eat for health (*p* <0.05). Those living in areas with less socioeconomic deprivation were more likely to speak about the characteristics of healthy food (*p* <0.05) and the benefits of eating for health (*p* <0.01).

**Table 5:** Results of Pearson *χ*2 test showing the significance of patterns of response elicited by the four prompt-terms

		Prompt-term used ^a^					Gender			Deprivation level ^b^			Age		
		Healthy eating	Eating for health	Balanced diet	Nutritional balance	*χ* ^*2*^	Male	Female	*χ* ^*2*^	L 1-3	L 4-7	*χ* ^*2*^	Less than 45	45 and older	*χ* ^*2*^
		n	n	n	n	*(df =3)*	n	n	*(df =1)*	n	n	*(df =1)*	n	n	*(df =1)*
1	Foods considered to be healthy	166^c^ (157)	169 (172)	176 (163)	128 (148)	8*	315 (335)	324 (304)	5*	219 (222)	420 (417)	*0*	377 (396)	262 (243)	4*
2	Foods that should be avoided	53 (39)	55 (43)	33 (41)	18 (37)	22***	95 (83)	64 (76)	*4+*	38 (55)	121 (104)	9**	77 (98)	82 (61)	14***
3	Descriptions of macronutrients	14 (29)	16 (32)	46 (30)	43 (28)	36***	56 (62)	63 (57)	*2*	51 (41)	68 (78)	*4*	83 (74)	36 (45)	*3*
4	Characteristics of healthy foods	19 (23)	26 (25)	22 (24)	26 (22)	*2*	58 (49)	35 (44)	4*	42 (32)	51 (61)	5*	65 (58)	28 (35)	*3*
5	Balance and moderation	22 (20)	21 (22)	17 (21)	23 (19)	*2*	41 (44)	42 (40)	*0*	29 (29)	54 (54)	*0*	54 (51)	29 (32)	*0*
6	The importance of lifestyle	24 (20)	18 (22)	22 (20)	16 (19)	*2*	47 (42)	33 (38)	*1*	29 (28)	51 (52)	*0*	57 (50)	23 (31)	*3*
7	Benefits of doing it	7 (11)	21 (12)	5 (12)	13 (11)	12**	21 (24)	25 (22)	*1*	26 (16)	20 (30)	10**	30 (29)	16 (18)	*0*
8	Importance of micronutrients	8 (11)	14 (12)	8 (11)	14 (10)	*4*	20 (23)	24 (21)	*1*	14 (15)	30 (29)	*0*	30 (27)	14 (17)	*1*
9	Reasons not to do it	13 (10)	10 (11)	10 (10)	8 (10)	*1*	21 (22)	20 (20)	*0*	8 (14)	33 (27)	4*	27 (25)	14 (16)	*0*
10	Diet and weight management	7 (8)	9 (8)	4 (8)	11 (7)	*4*	18 (16)	13 (15)	*0*	10 (11)	21 (20)	*0*	24 (19)	7 (12)	*3*
11	Food groups	1 (4)	1 (5)	3 (5)	13 (4)	*-†d*	10 (9)	8 (9)	*0*	4 (6)	14 (12)	*1*	16 (11)	2 (7)	6*
12	Reasons to have to do it	3 (3)	3 (4)	5 (4)	3 (3)	*-†d*	9 (7)	5 (7)	*1*	5 (5)	9 (9)	*0†e*	10 (9)	4 (5)	*1*
13	Nothing came to mind…	1 (2)	2 (2)	1 (2)	3 (2)	*-†d*	5 (4)	2 (3)	*1†e*	2 (2)	5 (5)	*0†e*	4 (4)	3 (3)	*0†e*
14	I don’t do it	0 (2)	5 (2)	0 (2)	0 (1)	*-†d*	6 (3)	0 (3)	6†e *	3 (2)	3 (4)	*1†e*	1 (4)	5 (2)	5†e *
15	It’s just something I do	1 (1)	1 (1)	0 (1)	1 (1)	*-†d*	2 (2)	1 (1)	*0†e*	1 (1)	2 (2)	*0†e*	1 (2)	2 (1)	*1†e*
16	It’s very important	1 (1)	2 (1)	0 (1)	0 (1)	*-†d*	3 (2)	0 (1)	*3 e*	1 (1)	2 (2)	*0†e*	2 (2)	1 (1)	*0†e*

Also illustrates the impact of gender, level of socioeconomic deprivation and age Expected counts shown are shown in brackets

df, degrees of freedom

^a^ Prompt-terms: HE, *Healthy eating* EfH, *Eating for health* BD, *Balanced diet* NB, *Nutritional balance*

^b^ Calculated from Carstairs DepCat scores (Level 1 is least deprived and Level 7 is poorest) [35]

^c^ Actual distributions are significantly different from expected distributions using Pearson’s *χ* ^2^ test: * *p* <0.05, **p <0.01, ***p <0.001, + *p* = 0.05

*†* Expected count is less than 5 in some cells, therefore Pearson’s *χ* ^2^ test not valid

^d^ Not a 2x2 table therefore Fisher’s exact test cannot be used

^e^ 2x2 table therefore Fisher’s exact test used

The prompt-term used produced different patterns of responses, when compared with the expected distributions, in four themes: foods perceived to be healthy (*χ*^2^ = 8, df = 3, *p* <0.05); foods to avoid (*χ*^2^ = 22, df = 3, *p* <0.001); descriptions of macronutrients (*χ*^2^ = 36, df = 3, *p* <0.001) and benefits of eating healthily (*χ*^2^ = 12, df = 3, *p* <0.01). *Healthy Eating* was associated with foods that are thought to be healthy and foods to avoid, *Eating for Health* was more associated with foods to avoid and the benefits of doing it, *Balanced Diet* was more associated with foods to avoid and descriptions of macronutrients and *Nutritional Balance* was more associated with descriptions of macronutrients and the benefits of eating healthily (Table 5). *Eating for Health* was the only prompt-term to elicit the response ‘I don't do it’ (*n* = 5) although there were too few expected responses in this category to allow *χ*^2^ testing.

Initially, 60 % of the focus group participants perceived no difference between the four prompt-terms. Further discussion led to the emergence of differences: *Eating for Health* was perceived as associated with actively managing an existing medical condition, *Healthy Eating* as a more general, “passive” term, *Balanced Diet* was perceived as old fashioned, associated with dieting for weight loss, while *Nutritional Balance* was perceived to be associated with sports people and those seeking to maximise their physical performance (Table 4).

There were no suggestions for other terms that might better communicate concepts linking health, food and the diet. A recurrent theme across all four focus groups was that messages intended to promote ‘healthy eating’ should focus on the positive rather than telling people what they should not eat; summarised succinctly by one participant: *“I think people get turned off by negativity, if you want to motivate people they’d rather hear ‘you're awesome, be more awesome’ not ‘you’re rubbish, be less rubbish”* (Focus group participant D2)

### Research question 3: What are public perceptions of government policies designed to promote health via the diet and foods, including product reformulation?

The focus groups were used to explore people’s perceptions of government interventions to promote health via the diet and foods. Awareness of nutritional information sources such as the Eatwell plate was very low, with only one person saying they had seen it before. This is supported by cross-referencing the responses in the word-association exercise with the Food Standards Agency (FSA) 8 tips for ‘healthy eating’ (Table 6) [39]. Only the ‘eat lots of fruit and vegetables’ message seems to be understood widely.

**Table 6:** Comparison of word-association responses against FSA’s tips for eating well from the Eatwell plate leaflet [39]

FSA 8 tips for eating well	Word-association related responses	Frequency	
		%	*n*
Base your meals of starchy foods	Carbohydrates, complex carbohydrates, pasta, cereals, whole grains, bread, wholemeal bread, brown bread	5	67
Eat lots of fruit and vegetables	Fruit, vegetables, fruit and vegetables, 5-a-day	22	304
Eat more fish, including one portion of oily fish per week	Fish, oily fish sardines	2	28
Cut down on saturated fat and sugar	Low fat, unsaturated fat, no fat, low sugar, no sugar	3	44
Try to eat less salt	Low salt, no salt	1	7
Get active and try to be a healthy weight	Exercise, diet and weight management	5	75
Drink plenty of water	Water, hydration	4	60
Don’t skip breakfast	Breakfast	1	6
Total		43	591

Participants in all 4 focus groups thought that the Eatwell plate leaflet had limited usefulness in terms of the way the information is presented and the level of interest in the content. They struggled to see how they would use the information to translate into a nutritionally balanced diet when used in isolation for an individual food choice *“I think it’s quite difficult, you know it looks nice on a plate, the idea’s good. But if you have a sandwich for lunch, I think it’s very hard to portion that out. You know, a cheese and tomato sandwich, is that right or wrong?”* (Focus group participant C2).

Four participants reported actively looking at food labelling, but only through concern about a particular issue, for example looking at fat and calories when trying to control their weight, or salt content if they had high blood pressure.

Finally, views on product reformulation were mixed. All four focus groups felt that the government ought to act to improve the ‘healthiness’ of products created and sold by the food industry, particularly to benefit children who would not notice changes in food composition. However, this was discussed in the context of reducing fat, salt and sugar, rather than seeking to improve the overall nutrient quality of foods. There was also an acknowledgement that acceptability and freedom of choice could not be ignored. Even if foods were reformulated people still had the freedom to buy less healthy options or consume more. *“Then we’d eat two biscuits instead of one cause there’s less calories.”* (Focus group participant C2)

## Discussion

The public is confronted by a plethora of similar messages related to health, food and diet daily, from a range of sources of varying authenticity and credibility [15–17]. Some are issued from well-informed sources aiming to improve long-term health by overall dietary improvement; others are arguably directed at short-term sales of food products without any attempt at achieving overall nutritional balance [15, 16]. Modifying diet composition has no immediate, objective, effects on health which are detectable by consumers, and a secondary industry ridiculing health messages has developed in some media sectors [18, 19]. Nutritionists are regularly pilloried as confused and constantly changing their advice [40, 41] and while trained nutritionists offer very constant, evidence-based dietary advice [15, 42], the media encourages unregulated non-evidence-based advice from prominent but untrained publicity seekers [15, 16]. The desensitisation of consumers to ‘healthy eating’ messages is unsurprising and to some extent may be triggered by the food industry itself [16, 18, 19, 43]. With this backdrop, it becomes important to ask exactly how consumers interpret the messages they meet [24].

### What do common terms used for health promotion, focusing on diet and foods, mean to consumers?

While many consumers have heard the health promotion messages focussing on food and diet enshrined in current dietary guidance, this does not necessarily mean they understand what constitutes a nutritionally balanced diet, as defined by the FSA’s eight tips for eating well (from the EatWell plate leaflet) (Table 6) [39], nor does it influence consumer behaviour towards eating more healthily. In the present study we report a partial, fragmented grasp of what it means to eat for health. The responses in both the word-association exercise and focus groups revealed an emphasis on foods perceived to be healthy and unhealthy, and controlling intake for weight management or management of a pre-existing medical condition, rather than a consideration of how to achieve an overall balance of nutrition in the diet. This is consistent with the findings of the two international systematic reviews of studies in this area, which found that the popular expression ‘healthy eating’ is polysemous and does not convey the notion of long-term influence on health [25, 26]. This study also reveals new responses to concepts linking health, food and the diet, not found in previous studies, specifically where participants had negative reactions to the concept(s), often arising from feelings of guilt about not conforming to what is perceived to be a ‘healthy’ diet or actively refusing to eat ‘healthily’, suggesting desensitisation and resistance to the ubiquitous healthy eating messages.

Public perceptions of messages conveying concepts linking health, food and the diet its role in the public ability to translate dietary guidance into behaviours underpinning lifelong health remains a relatively unexplored area. Freedhoff highlights the complex relationship played between public health and industry, and the use of health promotion messages for commercial purposes [18]. Beyond the sale opportunity, the lack of consistency in the use of the health message impacts on clarity and credibility of messages.

### Do these terms convey different meanings, and are there other terms that would communicate the concepts linking diet, food and health more clearly?

Not only did the language used affect how people perceived concepts linking health, food and the diet, but also the way in which the perceptions were elicited, ie: whether they were introduced as part of the word-association exercise or the focus groups (Table 7). All four prompt-terms tested elicited a different set of responses, in both the word association exercise and the focus groups. None of the terms fully conveyed the sense of eating a nutritionally balanced diet which supports health and fitness, reduces the risk of NCDs and may even have beneficial or therapeutic effects [44]. None of the focus group participants were able to suggest a term or language that would be more understandable, largely because of the confusion over what is meant by food and dietary behaviours underpinning lifelong health, a complex, multifactorial concept.

**Table 7:** Summary of differences in perceptions of the four prompt-terms arising from different methodologies used during the study

	Stage 1	Stage 2	Stage 3
	Pilot phase	Word-association^a, b^	Focus groups^c^
Healthy eating	A lifestyle choice – you chose to make a long term commitment	↑ Foods thought healthy *	A general term, passive (24 %)
		↑ Foods to avoid ***	
Eating for health	You have to do it, for a medical condition or to lose weight	↑ Foods to avoid ***	A proactive decision, due to medical condition (41 %)
		↑ Benefits of doing it **	Hard work and worthy
Balance diet	Everything in moderation – can have a treat today if you’re good tomorrow	↑ Foods thought healthy *	Old fashioned
		↑ Macronutrients ***	More about dieting and weight loss (30 %)
Nutritional balance	Technical term, boring and uninteresting	↓ Foods thought healthy *	More modern
		↓ Foods to avoid ***	Specifically for people involved in sports (12 %)
		↓ Macronutrients ***	

^a^ ↑ ↓ Indicates whether the number of responses for each theme was higher (↑) or lower (↓) than the expected count in the *χ* ^2^ test of association with levels of significance * *p* <0.05, ** *p* <0.01, *** *p* <0.001

^b^ Themes with fewer responses could not be tested for statistical significance as fewer than 5 expected counts

^c^ Initially 60 % of focus group participants (*n* = 10) saw little difference between the four terms. These differences emerged following group discussion

### What are public perceptions of government policies designed to promote health via the diet and foods, including product reformulation?

This study suggests that current government interventions such as the FSA’s Eatwell Plate were not well known or understood. They were thought to have limited usefulness in understanding how to eat for health both within a meal and over a period of time. Product reformulation by the food industry was seen as helpful, particularly for the next generation of children.

Providing nutritional information or telling consumers what they should or should not eat is not sufficiently effective in changing eating behaviour. Several theoretical models have been proposed for generating behavioural change. Amongst them, the trans-theoretical model [13], the theory of reasoned action [12], the health belief model [11] and the behaviour change wheel [14], all suggest that at least 3 components must be in place for behaviour to change: capability, opportunity and motivation. This study demonstrates that the language currently used to communicate concepts linking health, food and the diet is failing to convey the intended message: misperceptions reduce both the opportunity and motivation for behaviour change.

The findings in this study suggest that there is an opportunity to radically change both the message and method of delivery of health (promotion) messages focussing on food and diet, from public health agencies, the media and the food industry. There is no such thing as a typical consumer and consequently ‘one size fits all’ nutritional health promotion messages may remain ineffective, [45, 46], their constant repetition resulting in the desensitisation and negativity, as seen in this study, evidenced by the negative themes ‘*reasons not to do it*’, ‘*nothing came to mind*’ and ‘*I don’t do it*’. It should be feasible to categorise groups of consumers, as marketers do, and tailor nutritional health promotion messages and their delivery accordingly [47]. Such a categorisation should take into account levels of awareness, interest, motivation, capability, culture, gender, level of education, and socioeconomic status. Such an approach could be the basis for a unified model of intervention, as used in the North Karelia project in Finland [48].

It has been claimed that nutrition education cannot work and an ‘unobtrusive’ reformulation strategy, improving the nutritional content of food products without announcing them as ‘healthy’ options, may be the answer [49]. However such a strategy, designed to force compliance among consumers, would work to promote a healthful overall diet only if people had no choice about the combination of products and produce they consumed – they do have that choice. Strategies designed to improve the effectiveness public health campaigns in this area need to include clear communication of concepts linking health, food and the diet using appropriate language and delivery methods, in combination with interventions to improve the physical access to healthier choices in schools, supermarkets, workplaces etc. and the provision of healthier options by industry.

### Strengths and limitations

The main strength of this study lies with the number of strategies deployed in the design and analysis to maximise the trustworthiness of the results. The use of a mixed methods approach was appropriate for the complex research question being asked [50] and allowed triangulation of the results to improve levels of credibility and confirmability [51]. The large sample size in the semi-quantitative word-association exercise produced results with a confidence interval of 6 with 95 % confidence level [52]. Finally the clear inclusion criteria eliminated any potential participant bias arising from a background in nutrition or healthcare.

A key limitation was the low level of participation in the focus groups. This was partly explained by the choice to exclude participants sensitised to the subject (background in nutrition or health) and the lack of interest in participating from individuals approached. This refusal to participate is interesting in itself, and may mean that some of the more negative perceptions were under-represented in our results. Nevertheless, despite a high degree of consistency in the themes arising from the groups, it is unlikely that theoretical saturation was reached and key themes may not have been identified. .

The present study was conducted in Scotland, a country currently without legislative capacity to regulate food trade, with well-publicised associations between chronic disease and social deprivation related to poor diet quality [53]. However the issues addressed are global, and there is homogeneity with the findings of studies carried out internationally, so our findings may be more widely generalisable.

## Conclusion

Public perceptions of what it means to eat for health are influenced by the language used to communicate the concept and how it is communicated, resulting in confusion and misperceptions. Repeated use of non-specific messages and communication channels has resulted in wide scale desensitisation and antipathy among consumers.

Promoting dietary behaviour change in relation to lifelong health is a complex multifactorial problem, requiring prompting of the appropriate motivation, capability, and opportunity for change at individual level. Humans have evolved with an astonishing capacity to survive on a wide range of nutritional intakes, but there is an optimal range, and a health price is paid if we live outside that range. The amount of preventable ill-health associated with sub-optimal diet compositions is colossal and rising as a contributor to premature chronic diseases now that communicable diseases have been curtailed.

There are significant potential benefits associated with changing the general public’s eating habits – for both the long-term health of individuals and for reductions in the economic consequences of disability and premature death from NCDs. Only by investing the requisite resources into a tailored approach designed to meet the needs of specified groups of consumers can we build the necessary levels of commitment and reap these rewards.

### Ethical information

This study was conducted according to the guidelines laid down in the Declaration of Helsinki and all procedures involving human subjects were approved by the University of Glasgow College of Medicine, Veterinary Medicine and Life Sciences (MVLS) Ethical Committee.

## Additional files

Additional file 1:
**Focus group topic guide.**
Additional file 2
**Demographic characteristics of study participants.**

## Abbreviations

NCDs: Non-communicable diseases
FSA: Food standard agency
UK: United Kingdom
