# Supplementary material for: ‘Language is the source of misunderstandings’–impact of terminology on public perceptions of health promotion messages
Source: BMC Public Health. 2015 Jun 23;15:579. doi: 10.1186/s12889-015-1884-1 (PMC4476206; doi:10.1186/s12889-015-1884-1)
Supplement: Additional file 1: — Focus group topic guide. [file 12889_2015_1884_MOESM1_ESM.pdf]

Supplementary Table 1 Descriptive characteristics of the study population taking part in the word-association exercise

	Total		Edinburgh		Glasgow	
	%	n	%	n	%	n
**Total**	100	270	23	63	76	207
**Gender**						
Male	53	144	41	26	57	118
Female	47	126	59	37	43	89
**Age group**						
Under 45	63	169	64	40	62	129
45 and older	37	101	36	23	38	78
**Deprivation level**[1]						
Depcat 1-3[2]	34	93	64	40	26	53
Depcat 4-7[2]	63	177	36	23	74	154
**Median values**	Median	Range	Median	Range	Median	Range
Age	36	69	37	58	36	69
Deprivation level	4	6	3	6	4	6

[1] Calculated from Carstairs DepCat scores [24]

[2] Level 1 is the most affluent area and level 7 the poorest

Supplementary Table 2 Descriptive characteristics of the study population taking part in the focus groups

	**Total**		**Group A** (Glasgow)		**Group B** (Edinburgh)		**Group C** (Glasgow)		**Group D** (Glasgow)	
	%	n	%	n	%	n	%	n	%	n
**Total**	100	17	100	5	100	4	100	5	100	3
**Gender**										
Male	35	6	60	3	50	2	0	0	33	1
Female	65	11	40	2	50	2	100	5	67	2
**Age group**										
Under 45	59	10	60	3	100	4	0	0	100	3
45 and older	41	7	40	2	0	0	100	5	0	0
**Deprivation level**[1]										
Depcat 1-3[2]	18	3	0	0	25	1	40	2	0	0
Depcat 4-7[2]	82	14	100	5	75	3	60	3	100	3
**Median values**	Median	Range	Median	Range	Median	Range	Median	Range	Median	Range
Age	42	44	44	28	34	23	55	11	28	7
Deprivation level	5	6	5	2	7	4	4	5	6	2

[1] Calculated from Carstairs DepCat scores [24]

[2] Level 1 is the least deprived area and level 7 the poorest
