# Supplementary material for: ‘Language is the source of misunderstandings’–impact of terminology on public perceptions of health promotion messages
Source: BMC Public Health. 2015 Jun 23;15:579. doi: 10.1186/s12889-015-1884-1 (PMC4476206; doi:10.1186/s12889-015-1884-1)
Supplement: Additional file 2 — Demographic characteristics of study participants. [file 12889_2015_1884_MOESM2_ESM.docx]

**Supplementary file 1**

**Focus Group: High level topic/question guide**

**Aim: To understand perceptions of what ‘eating for health’ means and what alternative terminology could be used**

- Collection of informed consent

Ask participants to complete pre-session questionnaire – to establish key demographics (not names) ie: age, post code

**Ground rules and opening question**

- Welcome
- Introduction of researcher as facilitator, and role & input of the facilitator in the focus group
- Recording, anonymity, naming convention and questions

1. Ice breaker about TV show Saturday Kitchen? So familiar with the concept of food heaven and food hell?

**Introductory/transition**

1. In this discussion group we are talking about food and health – is this something you think about when deciding what to eat?
2. How important do you think what you eat is for your health? Now and in the future?

**Key questions**

1. Supermarkets and restaurants often brand products and menu items as ‘healthy’ options (use packaging as a prop), what do you think such terms mean – there any difference between them. Does this influence whether or not you would choose to buy/eat them?
2. A number of methods have been developed to try to encourage people to eat for their health. Are you aware of each and what do you think of them? Do you/would you find them helpful?

- Nutritional information/education eg: Eatwell plate? – use the brochures
- Dietary goals/guidelines eg: food labeling – use the food packaging
- Changing the nutritional content of food eg: salt reduction, use ingredient list from retailed food

1. A number of different terms are used to convey the principle of eating for your health eg: ‘eating for health’ ‘healthy eating’ ‘balanced diet’ and ‘nutritional balance’ Do these mean different things? In what way?

**Ending questions**

1. The main aim of this study is to understand how people perceive ‘eating for health’ and what the term means. Is there another term that would be more meaningful to you?
2. Is there anything you would like to add that you don’t think we have covered?

**Thank you and close…**
